# Use of pre-packaged chloroquine for the home management of presumed malaria in Malagasy children

**DOI:** 10.1186/1475-2875-5-79

**Published:** 2006-09-14

**Authors:** Arsène Ratsimbasoa, Milijaona Randrianarivelojosia, Pascal Millet, Jean Louis Soarès, Leon Rabarijaona, Benjamin Rakotoson, Denis Malvy, Didier Ménard

**Affiliations:** 1 Malaria Unit Research, Institut Pasteur de Madagascar, BP 1274, Antananarivo 101, Madagascar; 2 Epidemiology Unit, Institut Pasteur de Madagascar, BP 1274, Antananarivo 101, Madagascar; 3 EA3677, Centre René Labusquière, Université Victor Segalen Bordeaux 2, 146 rue Léo Saignat, 33076 Bordeaux Cedex, France; 4 Service de Santé de District de Moramanga, Toamasina, Madagascar

## Abstract

**Objective:**

The main objective of this study was to assess the quality of home malaria management with pre-packaged chloroquine in two areas in the Moramanga district of Madagascar. The knowledge, attitude and practices of care providers in terms of home treatment options were evaluated and compared. The availability of treatment options by studying retailers and community-based service providers was also investigated.

**Methods:**

A cross-sectional investigation in two communities, in the hamlets and villages located close to carers, retailers, community-based service providers and primary health centres was carried out.

**Results:**

Carers in the two districts were equally well aware of the use of pre-packaged chloroquine. Their first response to the onset of fever was to treat children with this antimalarial drug at home. The dose administered and treatment compliance were entirely satisfactory (100%) with pre-packaged chloroquine and rarely satisfactory (1.6% to 4.5%) with non pre-packaged chloroquine. In cases of treatment failure, the carers took patients to health centres. Chloroquine was supplied principally by private pharmacies and travelling salesmen selling unpackaged chloroquine tablets. Non pre-packaged chloroquine was the most common drug used at health centres. The frequency of positive rapid malaria tests (P = 0.01) was significantly higher in children treated with non pre-packaged chloroquine (38%) than in children treated with pre-packaged chloroquine (1.3%).

**Conclusion:**

Home malaria management should be improved in Madagascar. Efforts should focus on communication, the training of community-based service providers, access to pre-packaged drugs and the gradual withdrawal of pre-packaged chloroquine and its replacement by pre-packaged artemisinin-based combination therapies.

## Background

Malaria, known locally as "*tazo" *or "*tazomoka"*, is the leading cause of morbidity, mortality and hospital admission in Madagascar. Official data show a reported 2,114,400 cases of suspected malaria in 2003 (18.8% of all outpatient visits). About 740,000 of these cases occurred in children under the age of five years [1-3]. Limited physical access to public health facilities has been recognized to limit the provision of early treatment in developing countries, such as Madagascar. As a result of this limited access, communities resort to self-medication, through the unregulated private and informal sectors [4-6]. Thus, pharmacies, medicine shops or vendors, retail shops and medicines left over in homes are often the first source of treatment when symptoms begin [7]. As most of the children who die from malaria do so within 48 hours of the onset of illness, the early use of effective antimalarial medicines in or near the home can reduce the burden of malaria in endemic areas. This acknowledged time element is critical for saving children's lives in Africa and for reducing severe malaria morbidity and mortality in non-immune older children and adults living in other regions of the world [8,9]. A strong health care-delivery system should ideally provide early, reliable diagnosis and appropriate, prompt and effective treatment. However, most of those at highest risk of malaria in Madagascar live in rural areas geographically distant from health facilities (40% of people live more than five kilometres away from the nearest health facility, including 27% more than 10 kilometres away from the nearest health facility).

**Figure 1:**
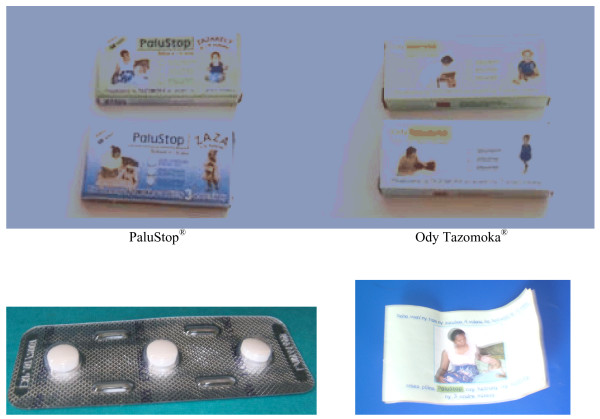
Pre-packaged chloroquine (PaluStop^® ^and Ody Tazomoka^®^) used for HMM in Madagascar (green boxes: for children aged 6 to 11 months, 3 pills of 75 mg, and blue boxes: for children from 12 to 59 months, 3 pills of 150 mg).

In 1989, Malagasy health policy-makers decided to recommend a strategy based on the home management of malaria (HMM). This decision followed a major malaria outbreak in the mid-1980s, with the widespread distribution of chloroquine (in the form of unpackaged 100 mg tablets). HMM was advocated as part of the National Malaria Control Programme (NMCP) in 1998. In November 2003, pre-packaged (PaluStop^®^) was introduced privately by an NGO called "Population Service International" and sold at an affordable price of US $0.025. Since March 2005, another form of pre-packaged chloroquine, Ody Tazomoka^®^, has been freely distributed at primary public health facilities. Both presentations of pre-packaged chloroquine are available for children from six to 11 months of age (three tablets of 75 mg) and for children from 12 to 59 months of age (three tablets of 150 mg) (Figure 1).

The main objective of this study was to assess the quality of HMM implementation with pre-packaged chloroquine, in two areas of the Moramanga district of Madagascar (in the periurban community of Ambohibary and in the remote rural community of Lakato). The knowledge, attitude and practices of caregivers in terms of home treatment options (non pre-packaged and pre-packaged chloroquine) were first evaluated and compared, at community level, by interviewing carers, and at primary health centre level, by interviewing the parents or guardians of children under the age of five years consulting for fever. The malaria burden among these children was also evaluated by rapid testing. The availability of treatment options in these two areas, by studying retailers and community-based service providers was also investigated.

## Materials and methods

### Study areas and setting

**Figure 2:**
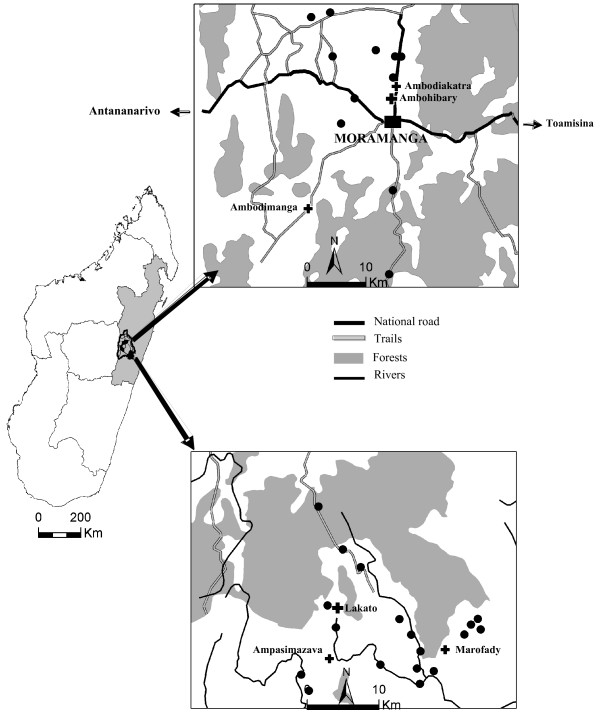
Map of the Moramanga District (with primary health centres).

This survey was conducted between April and June 2005, in two communities in the eastern foothills of the highlands of Madagascar: Ambohibary, a periurban community, and Lakato, a remote rural community (Figure 2).

Lakato (19°11'53.7" S, 48°23'32.6" E), is located in a remote area 40 kilometres away from the national road connecting Antananarivo, the capital of Madagascar, to Toamasina, the main harbour on the east coast. This area is poorly accessible, especially at the beginning of the study period (rainy season), and can only be reached by poor-quality tracks. It covers an area of 702 km^2 ^(altitude of 295 to 1,040 metres), with 15,831 inhabitants, living in 10 villages, with 44 hamlets. There is one "level two" primary health centre (CSB2: "*centre de santé de base niveau 2*", managed by a physician) and two "level one" primary health centres (CSB1: "*centre de santé de base niveau 1*" managed by a nurse). The prevalence of malaria in children under five years of age in this district has been estimated at 30%, with peaks in April-May and September-October (Malagasy *Ministère de l'Intérieur et Collectivité territoriale *for the district of Moramanga, 2005).

Ambohibary (18°54'55.5" S, 48°13'39.3" E) is located in a periurban area seven kilometres from the urban centre of Moramanga. It covers an area of 729 km^2 ^(altitude of 910 to 1,040 metres), with 16,557 inhabitants living in nine villages with 50 hamlets. It has one level 2 primary health centre and two level 1 primary health centres. The prevalence of malaria in children under five years of age in this district has been estimated at 27%, with a peak in April-May (data from the Malagasy *Ministère de l'Intérieur et Collectivité *for the district of Moramanga, 2005).

### Sampling methods and data collection

A cross-sectional investigation in the two communities, focusing on carers, drug sellers and community-based service providers and primary health centres in the hamlets and villages was carried out.

A two-level cluster sampling for the investigation of carers was used. A questionnaire was designed to collect data on knowledge, attitudes, practices and beliefs (KAPB) relating to malaria. This questionnaire was tested on 21 carers, to determine the frequency of knowledge relating to HMM. The minimum sample size required for the carer investigation was estimated at 392 (with 95% confidence intervals). Four hundred twenty carers (30 clusters of 14 carers) were finally included. All villages were informed one day before the investigation, by the head of the district. Each interviewer worked with a guide, who introduced him at each village. On the day of the investigation, the objectives of the study were explained to the children's carers before the interview. Carers were included in the study after they provided written informed consent. During the investigation, the interviewers were provided with questionnaires and pre-packaged chloroquine (PaluStop^® ^and Ody Tazomoka^®^). If the carers said that they had used pre-packaged chloroquine, they were asked to indicate the presentation used: PaluStop^® ^or Ody Tazomoka^®^. The investigator also checked whether insecticide-impregnated bed nets were used.

In Ambohibary and Lakato, all drug retailers and community-based service providers were interviewed with a designed questionnaire, after they had given written informed consent.

At primary health centres (three in Ambohibary and three in Lakato), all children under the age of five, clinically suspected of malaria (fever and recent history of fever) were enrolled and their parents or guardians were interviewed after they had provided written informed consent. Questionnaires were completed by health workers. The investigator carried out a rapid diagnostic test (OptiMal-IT^®^, DiaMed, Switzerland) to confirm the diagnosis of malaria and checked that the questionnaire had been completed correctly. The results of the rapid tests for malaria were communicated to the head of the primary health centre.

### Data analysis

Data were entered, processed and analysed with EpiInfo software (CDC, version 3.3.2). Chi-squared tests were used to assess the significant of differences between proportions. For continuous data, the significance of differences was assessed using Student's *t*-tests or Kruskal-Wallis tests.

## Results

The study was carried out in 27 hamlets and villages (13 in Ambohibary and 14 in Lakato). At the community level, 420 carers (196 in Ambohibary and 224 in Lakato), 10 community-based service providers (6 in Ambohibary and 4 in Lakato) and 32 retailers (22 in Ambohibary and 10 in Lakato) were interviewed. At primary health centre level, 341 patients attended health centres (109 in Ambohibary and 232 in Lakato), of which 207 children under the age of five, clinically suspected of malaria, were enrolled in the study (60 in Ambohibary and 147 in Lakato).

### Investigation of carers

More than 95% of the 420 carers interviewed were the mothers of the patients concerned. The youngest carer was 15 years old, the oldest was 65 years old and the median age of the carers was 29 years. Three-quarters (79.1% in Ambohibary and 69.2% in Lakato) of carers were married, or cohabited with a partner, the remaining carers being single, divorced, separated or widowed. The mean number of children under the age of five years looked after by an individual carer was significantly higher in Lakato (1.6) than in Ambohibary (1.4) (P = 0.0026). Significant differences were also found between the two districts in terms of professional activity (P < 10^-6^): 90.6% farmers in Lakato and 54.6% farmers in Ambohibary, with 19.4% craftsmen in Ambohibary and 0.4% in Lakato. Educational status profiles also differed significantly between Ambohibary and Lakato (P = 0.00005): no formal education (15.3% in Ambohibary and 42% in Lakato), primary education (51.5% in Ambohibary and 39.3% in Lakato), and secondary or further education (33.1% in Ambohibary and 18.8% in Lakato). The distance from the hamlet or village to the nearest health centre also differed significantly (P < 10^-6^) between Ambohibary and Lakato (Table 1). More than two-thirds of the carers in Ambohibary lived less than two hours walk from the nearest health centre, whereas more than two-thirds of the carers in Lakato lived more than two hours walk from the nearest health centre.

**Table 1:** The distances (walking times) between the villages and hamlets and the nearest health centre in the Ambohibary and Lakato areas (District of Moramanga, Madagascar)

	**Areas**		
		
**Distance between the villages and the nearest health centre (in time)**	**Ambohibary *n = 196 *% (CI95%)**	**Lakato *n = 224 *% (CI95%)**	***P <chi 2>***
0 to 30 minutes	29.1 (22.8–36)	11.6 (7.7–16.5)	< 10^-6^
30 to 60 minutes	35.7 (29–42.9)	4.5 (2.2–8.1)	
1 to 2 hours	13.8 (9.3–19.4)	7.1 (4.1–11.3)	
2 to 3 hours	20.9 (15.4–27.3)	40.2 (33.7–46.9)	
3 to 4 hours	0 (0–1.9)	14.3 (10–19.6)	
More than 4 hours	0.5 (0–2.8)	22.3 (17–28.3)	

The carers' knowledge about malaria was assessed (Table 2). Carers in Ambohibary were significantly better informed about malaria transmission than their counterparts in Lakato. In Ambohibary, carers were as familiar with PaluStop^® ^as with Ody Tazomoka^®^, whereas carers in Lakato were more familiar with PaluStop^®^, because this form of pre-packaged chloroquine was distributed freely in this area by and NGO.

**Table 2:** Carers' knowledge about malaria in the Ambohibary and Lakato areas (District of Moramanga, Madagascar)

		**Areas**		
			
**Variable**	**Results**	**Ambohibary**	**Lakato**	***P <chi 2>***
		**No (%)**	**No (%)**	
**Recorded symptoms**	Fever alone or in combination with others symptom*	195 (99.5)	223 (99.5)	NS
**Knowledge of malaria transmission**	By mosquitoes	144 (73.5)	73 (32.6)	< 10 ^-4^
	By other ways	23 (11.7)	23 (10.3)	NS
	None	29 (14.8)	128 (57.1)	< 10 ^-3^
**Malaria prevention used for children under 5 years**	Chloroquine	3 (1.5)	3 (1.3)	NS
	Impregnated bed nets	63 (35.2)	78 (34.8)	NS
	None	124 (63.2)	143 (63.8)	NS
**Knowledge of the use of pre-packaged chloroquine**	PaluStop^®^	140 (71.4)	171 (76.1)	NS
	Ody Tazomoka^®^	137 (69.9)	65 (29.0)	< 10 ^-6^

*Sweating, red eyes, shivering, joint pain, dizziness, cough, diarrhoea, runny nose, anorexia, vomiting

The management of uncomplicated childhood fever by carers in Ambohibary and Lakato is detailed in Table 3. In most cases, carers in both areas initially reacted to the onset of fever by treating the child with an antimalarial drug at home. However, in the rural area of Lakato, traditional treatment was widespread and used by more than one quarter of carers (bitter plants such as *Exacum sp*, from the Gentianaceae family, known as "*felatanandraka*" or "*aferontany*" in Malagasy). In most cases, the plant concerned was gathered, prepared and administered directly by the carer. The reasons for administering antimalarial drugs at home, according to carers, differed slightly between the two areas: (i) in Ambohibary, the first reason given by carers was that they already had such drugs at home (46.8% non pre-packaged chloroquine and 53.2% pre-packaged chloroquine), the second reason was that they had enough money to pay for a consultation with a health worker and the third reason was that the health centre was too far away; (ii) in Lakato, the two most important reasons given by carers were that they already had drugs at home (42.8% non pre-packaged chloroquine and 57.2% pre-packaged chloroquine), and the health centre being too far away. The third reason given was that there were often no health workers at the health centre. Regardless of the location, it was observed that the further the distance between the village and the health centre, the more likely the carer was to use antimalarial drugs at home (at Ambohibary, 66% for carers less than one hour from the health centre and 86% more than one hour from the health centre used antimalarial drugs at home; at Lakato, 83% of carers living less than one hour from the health centre and 97% living more than one hour from the health centre used antimalarial drugs at home). Conversely, the frequentation of health centres decreased with distance from the village, from 34% to 13% in Ambohibary and from 17% to 3% in Lakato.

**Table 3:** Carer's management of uncomplicated childhood fever in the Ambohibary and Lakato areas (District of Moramanga, Madagascar)

		**Areas**		
			
**Variable**	**Results**	**Ambohibary**	**Lakato**	***P <chi 2>***
			
		**No (%)**	**No (%)**	
**Action of the carer at fever onset**	Went to clinic/health centre	52 (26.5)	24 (10.7)	< 10 ^-5^
	Used antimalarial drugs at home (self-treatment)	139 (70.9)	138 (61.6)	0.05
	Used herbal medicines	5 (2.5)	57 (25.4)	< 10 ^-6^
	Went to traditional healer	0 (0)	5 (2.2)	NS

**Reasons given by carers for use of antimalarial drugs at home**	No health worker at health centre	0 (0)	46 (20.5)	< 10 ^-6^
	No money to pay for consultation	51 (25.7)	31 (13.8)	0.002
	Long distance to health centre	29 (14.6)	73 (32.8)	< 10 ^-4^
	Correct treatment known/Drugs already at home	117 (59.7)	74 (32.9)	< 10 ^-6^

**Antimalarial drugs used at home**	Not pre-packaged chloroquine	65 (46.8)	59 (42.8)	NS
	PaluStop^®^	50 (36.0)	2 (1.4)	NS
	Ody Tazomoka^®^	24 (17.2)	77 (55.8)	0.007

**Correct dose of chloroquine administered by carer**	Non pre-packaged chloroquine	1 (1.5)	1 (1.6)	NS
	PaluStop^®^	50 (100)	2 (100)	NS
	Ody Tazomoka^®^	24 (100)	77 (100)	NS

**Action after treatment failure**	Went to community-based service provider	7 (3.6)	24 (11)	NS
	Went to clinic/health centre	186 (94.9)	164 (73.0)	< 10 ^-6^
	Used herbal medicines	0 (0)	6 (3.0)	-
	Went to traditional healer	3 (1.5)	30 (13.0)	NS

In both areas, the dose administered and treatment compliance were entirely satisfactory (100%) with pre-packaged chloroquine treatment (PaluStop^® ^or Ody Tazomoka^® ^)and rarely satisfactory (1.6% to 4.5%) with non pre-packaged chloroquine treatment. The carers took a mean time of 3.5 days (3.7 in Ambohibary and 3.3 in Lakato) to evaluate the efficacy of treatment. In case of treatment failure (persistence of malaria symptoms), the carers in both communities took the patient to a health centre.

### Investigations of retailers and community-based service providers

Chloroquine was supplied principally by private pharmacies and travelling salesmen in the two areas. In the periurban area of Ambohibary and in the remote area of Lakato, health workers were responsible for educating carers, providing them with sufficient information to enable them to recognize the clinical symptoms of malaria, to assess its severity and to take appropriate action.

Unpackaged chloroquine tablets were sold more frequently than pre-packaged chloroquine tablets to carers by retailers and salesmen. The carers often bought an insufficient amount of chloroquine (two to four 100 mg tablets in Ambohibary and two to three 100 mg tablets in Lakato). A single tablet of 100 mg non pre-packaged chloroquine was sold at the same price as an entire box of pre-packaged chloroquine (full treatment for a child under five). Chloroquine often seemed to be in short supply in both areas.

Four retailers and salesmen in Ambohibary (18%) and two retailers in Lakato (20%) sold pre-packaged chloroquine (PaluStop^®^). This brand of chloroquine has been available for 12 months in Ambohibary and 10 months in Lakato.

Ody Tazomoka^® ^was distributed freely by the six community-based service providers in Ambohibary (100%) and by only one such service provider in Lakato (25%). This drug only became available a few weeks before the start of the study.

### Primary health centre investigations

Of the 207 children under five years of age seen at health centres (60 in Ambohibary and 147 in Lakato), 50% were initially treated at home in Ambohibary and 27.2% in Lakato. The mean time between the onset of fever and consultation at a health centre was two days in Ambohibary (0 to 6 days) and three days in Lakato (0 to 14 days).

In both areas, non pre-packaged chloroquine was the drug most frequently used for treatment at home (53.7% in Ambohibary and 50.8% in Lakato). Pre-packaged chloroquine was used less frequently (19.6% in Ambohibary and 10.4% in Lakato). Of the 70 children treated with chloroquine at home, 55 were treated with non pre-packaged chloroquine and 15, with pre-packaged chloroquine. Rapid tests for malaria were positive in 40% of cases in Ambohibary and 45% of cases in Lakato. The frequency of positive rapid tests for malaria (P = 0.01) was significantly higher for children treated with non pre-packaged chloroquine (38% positive rapid tests overall, 18% in Ambohibary and 52% in Lakato) than for children treated with pre-packaged chloroquine (1.3% positive rapid tests overall, 2% in Ambohibary and 0% in Lakato).

## Discussion

The success of health interventions such as HMM requires communities to have detailed knowledge of perceived health problems [10]. These health problems can only be recognized as amenable to modern health interventions if their manifestations are perceived as being amenable to modern treatment [11]. In Ambohibary and Lakato, malaria is the most common health problem in childhood. The prevalence of malaria in children under the age of five in the health centres of these two areas is similar to national estimates [2]. Thus, these communities are willing to participate in health interventions aiming to reduce the frequency of malaria in their children.

This study was carried out in two areas with different socio-demographic profiles, representative of the current situation in Madagascar. In these two areas, most of the carers were mothers, as reported in other areas of Madagascar [11] and in Africa [12,13]. The mother's ability to associate malaria with fever has important implications for the survival of her child in areas of endemic malaria [14,15]. In Ambohibary as well in Lakato, investigation of carers showed 99.5% of them identified malaria as fever alone or in combination with other symptoms such as sweating, red eyes, shivering, headache, joint pain, dizziness, cough, diarrhoea, runny nose, anorexia or vomiting. This frequency is higher than that reported in other studies: 85.6% in the Democratic Republic of Congo [16] and 80.8% in Burkina Faso [17].

In the two areas studied, mothers faced with a case of suspected malaria in one of their children chose first to administer antimalarial drugs at home, seeking care for their child at a health facility only in case of treatment failure. This shows that mothers have the potential to manage malarial fever correctly at home and to consult health workers. These positive attitudes and practices should be reinforced during health interventions, to decrease severe morbidity and mortality from childhood malaria by improving case management. However, even in Ambohibary, where the mothers knew that malaria was transmitted by mosquitoes, only one third of the children slept under insecticide-impregnated bed nets. Although many mothers were aware of pre-packaged chloroquine (69.9% in Lakato and 76.1% in Ambohibary) only half of them used it, often because they had the drug in an non-packaged form at home. This was the case in the periurban area of Ambohibary, where the flow of information about HMM strategy and the supply of pre-packaged chloroquine are easy to establish. In this area, either information about the HMM strategy was not transmitted by health workers due to a lack of motivation, or carers chose not to use the free pre-packed chloroquine because they thought that it was not effective. In the remote area of Lakato, the main reason for not using pre-packaged chloroquine was the time lag to the release of free pre-packaged chloroquine, because of a lack of co-ordination between the communities and the headquarters of the district health authority.

Chloroquine was introduced into Madagascar in 1945 and is the antimalarial drug best known to the Malagasy people [18,19]. As previously reported in Madagascar [11] and Nigeria [20], non pre-packaged tablets were not used appropriately in either Ambohibary or Lakato. In these two areas, the use of non pre-packaged chloroquine by carers led to a significantly higher rate of treatment failure, as shown in this study for health centre-based analyses. By contrast, all carers using pre-packaged chloroquine used this drug at the correct dose. For HMM to be effective, the population must be aware of: (i) the correct dose and (ii) the need to complete treatment (drug resistance often develops because the course of treatment is not completed). The easy-to-follow leaflet, with illustrations on the box and the blister-packed tablets may also have encouraged carers to use pre-packaged chloroquine. Similarly, in Uganda [21], mothers favoured pre-packaged chloroquine, known as "*homapak*", because it was neatly and attractively packaged. A decrease in the efficacy of chloroquine against *P. falciparum *has recently been recorded in several areas [22,23]. Based on these findings, the Malagasy Ministry of Health could improve HMM by withdrawing pre-packaged chloroquine, gradually replacing it with pre-packaged artemisinin-based combination therapies (ACT, such as artesunate plus amodiaquine, the first-line treatment recommended for uncomplicated malaria in Madagascar in the forthcoming revised national policy. ACT acts more rapidly, has a higher cure rate and causes fewer side-effects than other treatment, such as chloroquine, but is more expensive.

However, if pre-packaged drugs are to be used, efforts are required: (i) to ensure effective communication, favouring correct care-seeking behaviour and appropriate and effective HMM for the treatment of febrile illness. Particular attention should be paid to stressing the link between mosquitoes and malaria for less literate carers, and the importance of prompt and complete treatment with pre-packaged drugs; (ii) to train community-based service providers, to ensure that they have the necessary skills and knowledge to manage febrile illness or malaria. This training could take place within the commercial sector, but might then have to be based on a limited curriculum, dictated by the amount of time that trainers are willing to spend without compromising their own businesses; (iii) to supervise and monitor implementation activities at the community and health facility levels.

## Authors' contributions

AR, MR and DMe were involved in all stages of this study. PM, JLS and LR were involved in the design of the study. BR participated in the coordination of the field work. DMa helped to compose the manuscript and gave constructive advice.
